# Fermi Velocity Dependent Critical Current in Ballistic Bilayer Graphene Josephson Junctions

**DOI:** 10.1021/acsnanoscienceau.4c00080

**Published:** 2025-03-19

**Authors:** Amis Sharma, Chun-Chia Chen, Jordan McCourt, Mingi Kim, Kenji Watanabe, Takashi Taniguchi, Leonid Rokhinson, Gleb Finkelstein, Ivan Borzenets

**Affiliations:** † Department of Physics and Astronomy, Texas A&M University, College Station, Texas 77843, United States; ‡ Department of Physics, Duke University, Durham, North Carolina 27701, United States; ¶ Department of Physics and Astronomy, Purdue University, West Lafayette, Indiana 47907, United States; § Advanced Materials Laboratory, NIMS, Tsukuba 305-0044, Japan

**Keywords:** Graphene, Bilayer Graphene, Josephson Junctions, Fermi Velocity, Andreev Levels

## Abstract

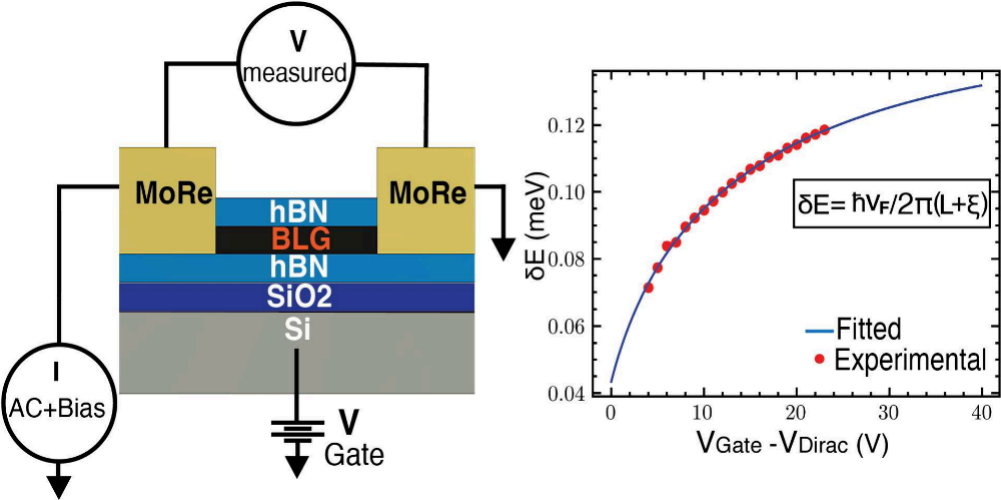

We perform transport measurements on proximitized, ballistic, bilayer graphene Josephson junctions (BGJJs) in the intermediate-to-long junction regime (*L* > ξ). We measure the device’s differential resistance as a function of bias current and gate voltage for a range of different temperatures. The extracted critical current *I*_C_ follows an exponential trend with temperature: exp(−*k*_B_*T*/*δE*). Here *δE* = ℏν_*F*_/2*πL*: an expected trend for intermediate-to-long junctions. From *δE*, we determine the Fermi velocity of the bilayer graphene, which is found to increase with gate voltage. Simultaneously, we show the carrier density dependence of *δE*, which is attributed to the quadratic dispersion of bilayer graphene. This is in contrast to single layer graphene Josephson junctions, where *δE* and the Fermi velocity are independent of the carrier density. The carrier density dependence in BGJJs allows for additional tuning parameters in graphene-based Josephson junction devices.

Ballistic graphene Josephson junctions (GJJs) have been widely utilized as a platform to study novel quantum physics phenomena^1,2^ and devices,^3^ including: entangled pair generation,^4,5^ topological states arising from the mixing of superconductivity and quantum Hall states,^6^ as well as photon sensing via bolometry/calorimetry.^7^ Superconductor–normal metal–superconductor Josephson junction (SNSJJ) hosts Andreev bound states (ABS), which carry supercurrents across the normal region of the JJ; in order to enter the ballistic regime, a disorder-free weak link and high transparency at the SN interface are necessary. Hexagonal Boron-Nitride (hBN) encapsulated graphene as a weak link enables highly transparent contacts at the interface while keeping graphene clean throughout the fabrication process.^8^ Here, we study proximitized, ballistic, bilayer graphene Josephson junctions (BGJJs). Bilayer graphene devices (in contrast to monolayer) allow extra potential tunability via a nonlinear dispersion relation, applied displacement field, or lattice rotation.^1^

The critical current (*I*_*C*_) of SNSJJ in the intermediate-to-long regime, where the junction length (*L*) ≥ superconducting coherence length (ξ_0_), scales with temperature (*T*) as *I*_*C*_ = exp(−*k*_B_*T*/*δE*). Here, *δE* = ℏν_*F*_/2*πL*, an energy scale related to the ABS level spacing.^2,9−13^ Note that in the intermediate regime (*L* ≈ ξ_0_) *δE* is found to be suppressed.^5^ A previous study of GJJs found that in this regime the relation was held more precisely when ξ was taken into account along with *L*, that is, *δE* = ℏν_*F*_/2π(*L* + ξ).^2,13^ Monolayer graphene displays a linear dispersion relation, which results in a constant Fermi velocity (ν_*F0*_). Thus, in ballistic GJJs, *δE* remains independent of the carrier density. In comparison, bilayer graphene displays a quadratic dispersion relation at low energies. In BGJJs we studied, a back-gate voltage (*V*_*G*_) controls the carrier density, and *δE* dependence on *V*_*G*_ is observed. Using *δE*, we extract the Fermi velocity in bilayer graphene: It is seen that ν_*F*_ increases with *V*_*G*_ and saturates to the constant value, ν_*F0*_, of the monolayer graphene.

Our device consists of a series of four terminal Josephson junctions (on SiO_2_/Si substrate) made with hBN encapsulated bilayer graphene contacted by Molybdenum–Rhenium (MoRe) electrodes. Bilayer graphene is obtained via the standard exfoliation method. It is then encapsulated in hexagonal boron-nitride using the dry transfer method.^14^ MoRe of 80 nm thickness is deposited via DC magnetron sputtering. The resulting device has four junctions of lengths 400, 500, 600, and 700 nm. The width of the junctions is 4 μm. The device is cooled in a Leiden cryogenics dilution refrigerator operated at temperatures above 1 K, and measurements were performed using the standard four-probe lock-in method. A gate voltage *V*_*G*_ is applied to the Si substrate with the oxide layer acting as a dielectric, which allows modulation of the carrier density.^2,5,6,15−17^Figure 1(a) displays the differential resistance (*dV*/*dI*) map of the 400 nm junction at *T* = 1.37 K; we see zero resistance (black region) across all applied *V*_*G*_ indicating the presence of supercurrent. As the bias current *I*_*bias*_ is swept from negative to positive values, the junction first reaches its superconducting state at a value |*I*_*bias*_ | = *I*_*R*_, known as the retrapping current. Then, as |*I*_*bias*_| is increased to higher positive values, the junction transitions to the normal state at |*I*_*bias*_| = *I*_*S*_, known as the switching current. Figure 1(a) shows that the junction can sustain a larger region of critical current as we modulate the carrier density to higher values via *V*_*G*_. Figure 1(b) displays line traces extracted from the *dV*/*dI* map which shows hysteresis in *I*_*R*_ and *I*_*S*_. This is a commonly observed phenomenon in underdamped junctions^15,18^ or can also be attributed to self-heating.^16,17,19^ The measured switching current *I*_*S*_ is slightly suppressed compared to the junction’s “true” critical current *I*_*C*_. However, previous measurements on the statistical distribution of *I*_*S*_ in similar graphene devices found that *I*_*S*_ is suppressed from *I*_*C*_ by no more than 10% for critical currents up to a few μA.^2,20−22^

**Figure 1:**
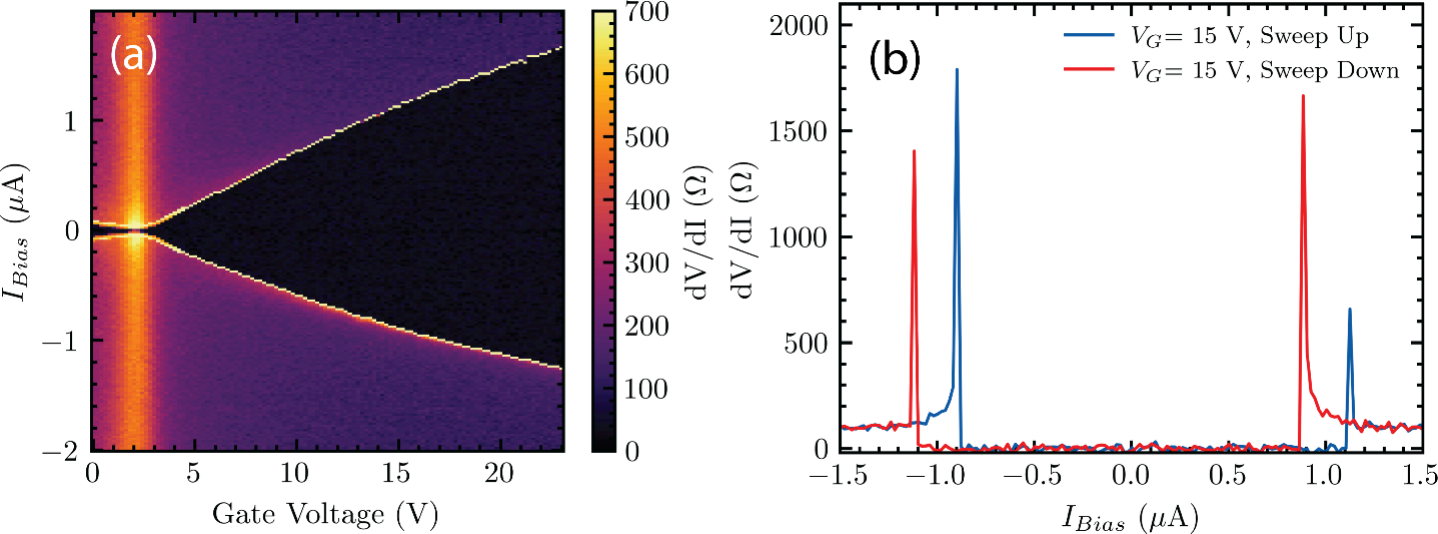
(a) Differential resistance (*dV*/*dI*) versus gate voltage (*V*_*G*_) and bias current *I*_*bias*_ taken at *T* = 1.37 K. The black region around zero bias corresponds to the superconducting state. *I*_*bias*_ is swept up (from negative to positive). Thus, the transition at negative bias corresponds to the retrapping current *I*_*R*_, while the transition at positive bias is the switching current *I*_*C*_. (b) Vertical line cut of the resistance map taken at *V*_*G*_ = 15 V, *T* = 1.37 K, showing the device’s *dV*/*dI* versus bias current. Blue line corresponds to *I*_*bias*_ swept up, with red line swept down (positive to negative).

Extracting the critical current *I*_*C*_ from the differential maps for different temperatures, we can see that *I*_*C*_ falls exponentially with inverse *T* (Figure 2c) We also extract the conductance of the junction in the normal regime (*I*_*Bias*_ ≫ *I*_*C*_). Figure 2(b) shows this conductance (*G*) for the 400 nm junction device. Due to the significant contact resistance (*R*_*C*_) of the device, the measured conductance *G* is uniformly suppressed compared to the ballistic limit expectation. However, when accounting for *R*_*C*_ within the fit, we find that the conductance *G* scales as the square-root (as opposed to linearly) of *V*_*G*_ (blue curve of Figure 2(b)). This is consistent with ballistic transport.^2,23^ To further demonstrate the ballistic nature of the device, we present normal resistances (*R*_*N*_) of junctions of length 500, 600, and 700 nm with the fitted, constant contact resistance *R*_*C*_ subtracted (Figure 2(b) inset). The inset plot shows that the values of *R*_*N*_ – *R*_*C*_ are independent of the junction length, demonstrating the ballistic nature of the devices.

**Figure 2:**
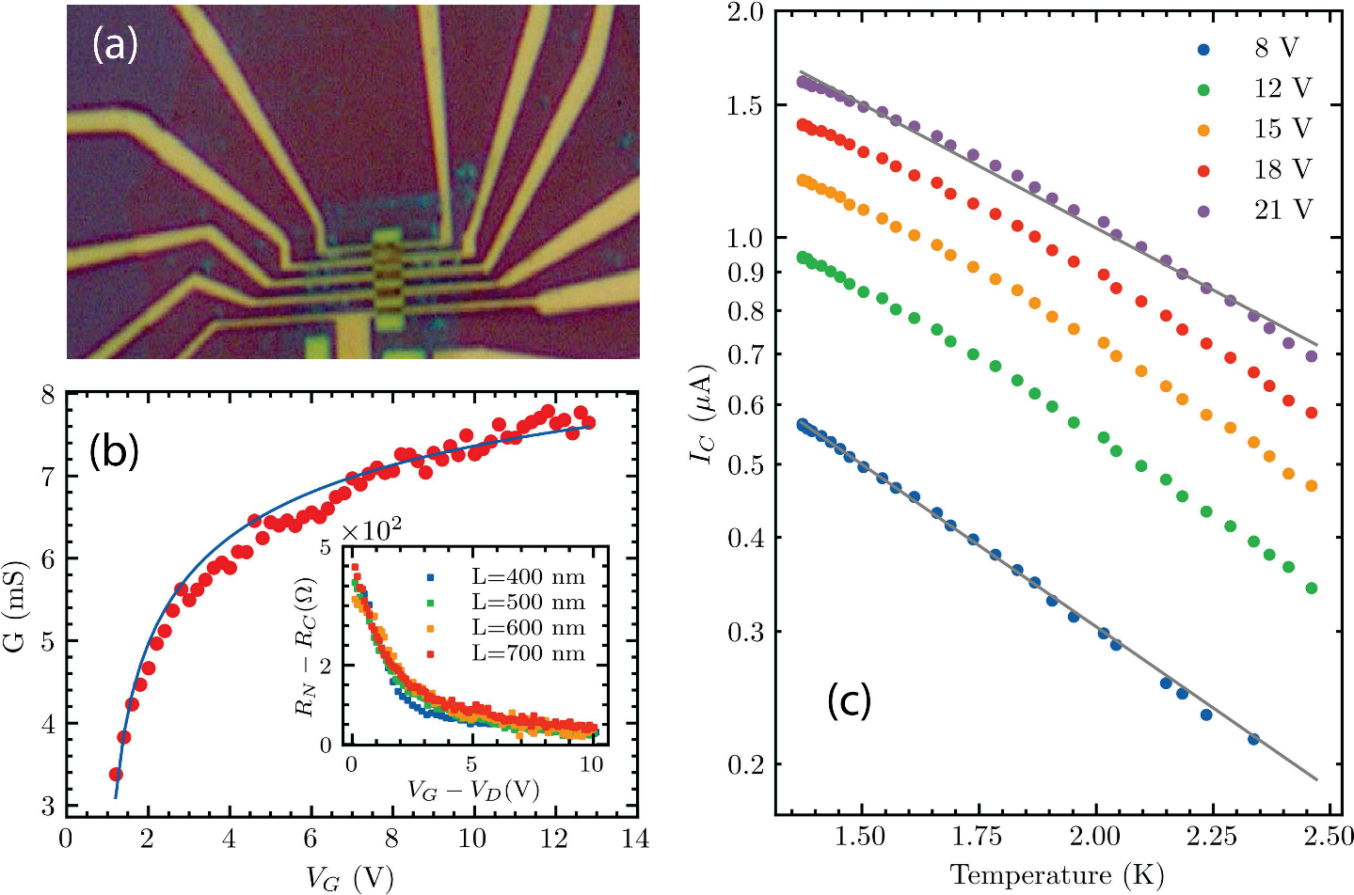
(a) Device picture. Image shows a series of junctions with different lengths: 400 nm, 500 nm, 600 and 700 nm. (b) The ballistic conductance vs gate voltage for *L* = 400 nm junction. The blue curve corresponds to the fit for ballistic devices, with an addition of a contact resistance. The inset shows junction resistance minus the parasitic contact resistance plotted against gate voltage from the Dirac point for all our devices. (c) Critical currents *I*_*C*_ of *L* = 400 nm junction plotted against temperature *T*, for various gate voltages, on a semilog scale. The plots show *V*_*G*_ dependence of *I*_*C*_: the gray lines show that the slope of the curve for the lowest plotted gate *V*_*G*_ = 8 V is smaller than the slope of the highest plotted gate *V*_*G*_ = 21 V.

To extract *δE* of the junction, we go to the discussion of *I*_*C*_ vs the temperature trends in Figure 2(c). Here, the *y*-axis is plotted in logarithmic scale. From the slope of the curves log(*I*_*C*_) = −(*k*_B_/*δE*)*T* for each gate, one can extract *δE* versus *V*_*G*_ (plotted in Figure 3a). Unlike for the case of monolayer graphene, a clear dependence on *V*_*G*_ is seen (The observed trend further supports the view that our devices operate in the long ballistic regime. Diffusive Josephson junctions are governed by the Thouless energy (22,24) which does not match the trend with respect to *V*_*G*_ seen in Figure 3(a)). The energy *δE* scales linearly with the Fermi velocity *v*_*F*_ (Figure 3(b)). Note that calculating *v*_*F*_ from *δE* for junctions in the intermediate regime requires knowledge of the superconducting coherence length ξ. In the fit discussed below, we use ξ’s dependence in *v*_*F*_.

**Figure 3:**
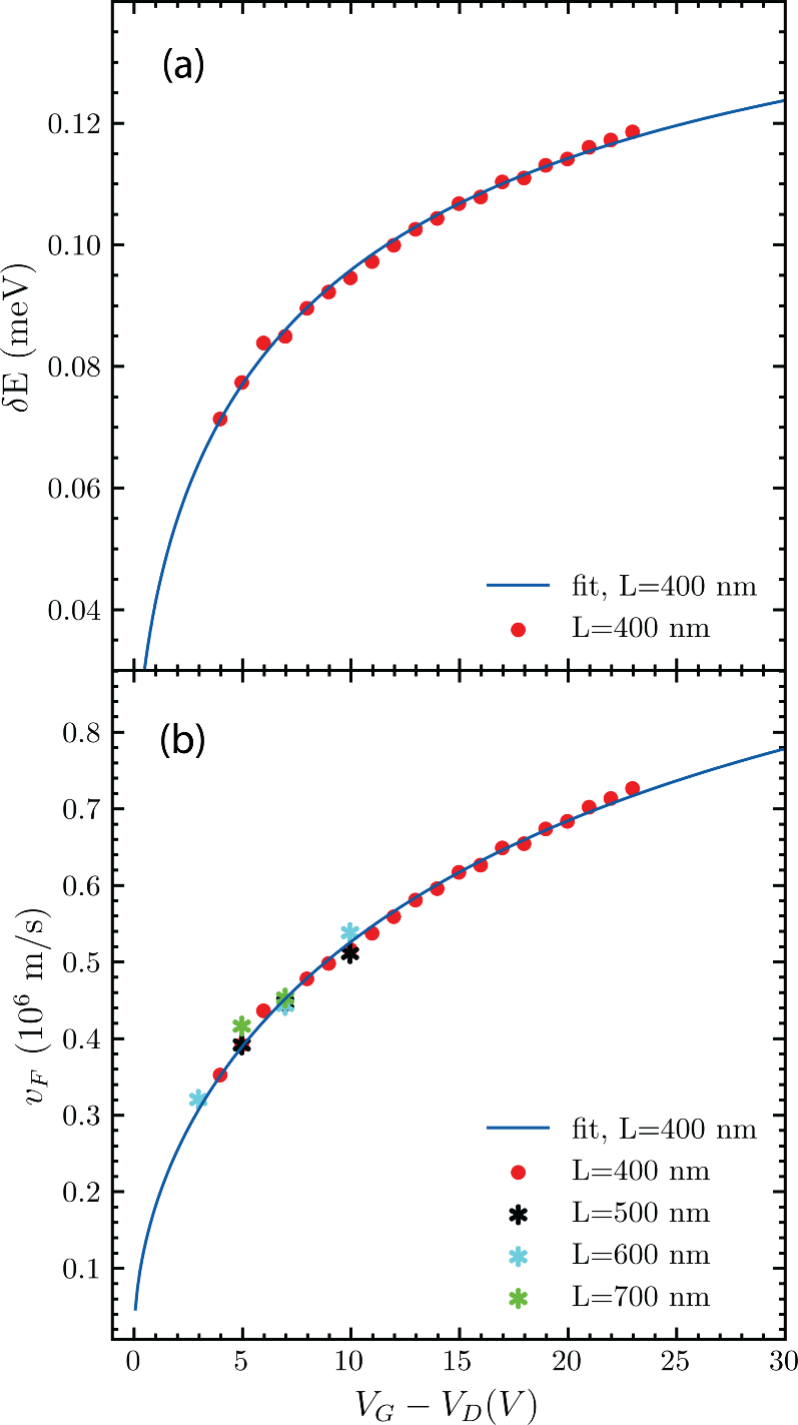
(a) Energy *δE* extracted from the slope of log(*I*_*C*_) vs *T* plotted against the gate voltage *V*_*G*_ from the Dirac point of the junction with *L* = 400 nm. We see *δE* dependence on the carrier density modulated via the gate voltage for the junction. (b) Fermi velocity (*v*_*F*_) calculated from *δE* using the device dimensions and parameters obtained from the fit to theory. The solid line represents the theoretical trend as fitted to the data for the *L* = 400 nm junction. In addition, panel (b) shows calculated *v*_*F*_ for the other junctions using parameters obtained from the *L* = 400 nm fit.

We now compare the experimentally obtained *δE* (and *v*_*F*_) to the theoretical expectation. With the dispersion relation for bilayer graphene written as , we get the expression for the Fermi velocity: .^25−27^ Here, γ_1_ = 0.39 eV a parameter describing the interlayer coupling,^25^*k* is the momentum wavevector, and m* is the effective mass of electrons. Moreover, the Fermi energy  for bilayer graphene scales as . The carrier concentration *n*, controlled by the applied gate voltage *V*_*G*_, is given by  with *V*_*D*_ as the gate voltage at the Dirac point. The total capacitance *C*_*Total*_ is a combination of quantum capacitance *C*_*q*_ and gate oxide capacitance *C*_*ox*_: . The quantum capacitance *C*_*q*_ for bilayer graphene is determined by , where *e* is the electron charge. The gate oxide capacitance per unit area is , where ϵ_0_ is the vacuum permittivity, ϵ_*r*_ is the relative permittivity of the oxide, and *d* is the thickness of the oxide layer. For a silicon oxide gate with *d* = 300 nm we get *C*_*ox*_ ≈ 115 μF/m^2^. Thus, the full expression for the Fermi velocity *v*_*F*_ is
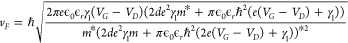
1Note that the effective mass m* typically ranges from 0.024 *m*_*e*_ to 0.058 *m*_*e*_ for 1 × 10^12^ ∼ 4 × 10^12^ carriers/cm^2^,^28^ where *m*_*e*_ is the electron rest mass. Experimental data provides us with the following: . We also note that ξ has a dependence on *v*_*F*_ and the superconducting gap Δ: ξ = ℏ*v*_*F*_/2Δ.^13^ To fit *δE*, the model is set as  where m*, Δ, *V*_*D*_, and *d* are the fitting parameters and *V*_*G*_ is the independent variable. (We use the as-designed length of the device *L* and take ϵ_*r*_ = 3.9 for SiO_2_.)

The resulting fits of the data from the 400 nm junction for *δE* and *v*_*F*_ are plotted as solid lines in Figure 3(a) and Figure 3(b) respectively. Moreover, taking the fitted parameters from Table 1, we calculate the Fermi velocity *v*_*F*_ for the available data points of all other junctions on the same substrate. As seen from Figure 3(b), the calculated *v*_*F*_ of all devices is in good agreement with the fit obtained from the 400 nm junction (this is as expected for devices on the same substrate as long as they have consistent parasitic doping and a superconductor–graphene contact interface). The fitted parameters are summarized in Table 1. All fall within the range of expected values, with Δ being consistent with previously measured values for graphene/MoRe junctions.^2^ Furthermore, using the values obtained from the model, we find that *v*_*F*_ saturates to the value of 1.1 × 10^6^ m/s as *V*_*G*_ tends to infinity.

**Table 1:** Fitting Parameters Used to Match the Measured *δE*, and Consequently the Fermi Velocity *v*_*F*_, versus Gate to the Theoretical Expectation Described in Equation 1^a^

Parameter	Fitted Value	Expected Value
Δ	0.99 meV	0.8 ∼ 1.2 meV
*d*	323 nm	280 ∼ 330 nm
m*	0.028 *m*_*e*_	0.02 ∼ 0.06 *m*_*e*_
*V*_*D*_	2.04 V	≈+2 V

a We see that resulting fitted values match closely to what is expected. The expected gate dielectric thickness *d* is estimated from the substrate specifications plus the bottom hBN thickness. The expected Dirac point voltage *V*_*D*_ is obtained from the resistance map. The expectations for superconducting gap Δ and the effective mass *m*_*i*_ are obtained from previous works.^5,28^

In conclusion, we study the evolution of the critical current with respect to the gate in bilayer graphene Josephson Junctions (BGJJs). Using the critical current-temperature relation expected for intermediate-to-long junctions, we extract the relevant energy scale *δE* and find that it has a clear gate dependence. As *δE* is proportional to the Fermi velocity *v*_*F*_ in bilayer graphene, we are able to match the observed gate dependence to the theoretical expectation. Our observation is contrasted with monolayer graphene JJs, which do not have a gate-dependent *δE*. This result showcases the greater tunability of BGJJs, and offers additional avenues for device characterization. Although not observed here, it should be possible to engineer Josephson junctions that transition from the short to the intermediate/long ballistic regimes in situ via gate voltage. The ability to tune ABS level spacing could have applications in self-calibrating sensors, or for matching resonance conditions in multiterminal superconducting devices.
