# Correction to “Fermi Velocity Dependent Critical Current in Ballistic Bilayer Graphene Josephson Junctions”

**DOI:** 10.1021/acsnanoscienceau.5c00093

**Published:** 2025-08-11

**Authors:** Amis Sharma, Chun-Chia Chen, Jordan McCourt, Mingi Kim, Kenji Watanabe, Takashi Taniguchi, Leonid Rokhinson, Gleb Finkelstein, Ivan Borzenets

A correction was made to the Acknowledgments. The revised Acknowledgments are shown below.

